# Dietary carotenoid intake and risk of developing preeclampsia: a hospital-based case–control study

**DOI:** 10.1186/s12884-022-04737-5

**Published:** 2022-05-21

**Authors:** Ting Kang, Yanhua Liu, Xi Chen, Xuemin Huang, Yuan Cao, Weifeng Dou, Dandan Duan, Yacong Bo, Stanislav Seydou Traore, Xianlan Zhao, Wenjun Fu, Fangfang Zeng, Jun Liu, Quanjun Lyu

**Affiliations:** 1 Department of Nutrition and Food Hygiene, College of Public Health, Zhengzhou University, Zhengzhou, 450000 Henan China; 2 Department of Nutrition, the First Affiliated Hospital of Zhengzhou University, Zhengzhou, 450052 Henan China; 3 The Third Affiliated Hospital of Zhengzhou University, Zhengzhou, 450052 Henan China; 4 Department of Clinical Nutrition, Luoyang New Area Peoples Hospital, Luoyang, 471023 Henan China; 5 Jockey Club School of Public Health and Primary Care, the Chinese University of Hong Kong, Hong Kong, 999077 China; 6 Department of Obstetrics, the First Affiliated Hospital of Zhengzhou University, Zhengzhou, 450052 Henan China; 7 Department of Epidemiology, School of Medicine, Jinan University, No.601 Huangpu Road West, Guangzhou, 510632 Guangdong China; 8 Department of Preventive Medicine, School of Public Health, Zunyi Medical University, Zunyi, 563006 Guizhou China

**Keywords:** Preeclampsia, Carotenoids, Chinese, Pregnant women, Case–control study

## Abstract

**Background:**

The effect of carotenoids on the risk of preeclampsia (PE) is uncertain. We aimed to examine the associations between the intake of dietary carotenoids and related compounds by pregnant women in China, and the risk of their developing PE.

**Methods:**

Four hundred and forty PE cases and 440 age- (± 3 years), gestational age- (± 1 weeks) and gestational diabetes mellitus status- (yes/no) matched healthy controls were recruited from March 2016 to June 2019. Dietary intake of carotenoids was assessed using a 79-item validated food-frequency questionnaire. Odds ratios (ORs) and 95% confidence intervals (CIs) were estimated using conditional logistic regression.

**Results:**

After adjusting for potential confounders, we found that the intake of total carotenoids, β-carotene, β-cryptoxanthin, lycopene, and lutein and zeaxanthin (lut-zea) were negatively associated with the odds of developing PE. Compared with the lowest quartile intake, the multivariate-adjusted OR (95% CI) of the highest quartile intake was 0.29 (0.16–0.54, *P*_trend_ <  0.001) for total carotenoids, 0.31 (0.16–0.58, *P*_trend_ <  0.001) for β-carotene, 0.50 (0.27–0.90, *P*_trend_ = 0.007) for β-cryptoxanthin, 0.55 (0.30–0.99, *P*_trend_ = 0.04) for lycopene and 0.32 (0.17–0.61, *P*_trend_ = 0.001) for lut-zea. However, no significant associations were observed between the risk of developing PE and α-carotene intake (OR = 0.75, 95% CI: 0.41–1.36, *P*_trend_ = 0.28). Moreover, similar negative associations were found for every one-standard-deviation increase in the intake of total carotenoids, β-carotene, β-cryptoxanthin, lycopene and lut-zea.

**Conclusion:**

These results indicate that a high intake of total carotenoids, β-carotene, β-cryptoxanthin, lycopene and lut-zea may be associated with a low risk of developing PE.

**Supplementary Information:**

The online version contains supplementary material available at 10.1186/s12884-022-04737-5.

## Background

Preeclampsia (PE) is a major pregnancy complication with a worldwide incidence rate of 2.61%, as reported by the World Health Organization [1]. PE occurs in 4–5% of pregnancies worldwide [2] and leads to 75,000 maternal deaths each year [3]. It is also one of the main causes of maternal, foetal and neonatal deaths in low- and middle-income countries [4]. The main clinical manifestations of PE are hypertension, proteinuria and oedema after 20 weeks of gestation, which in cases of severe PE may be accompanied by systemic multiple organ damage; heart failure; haemolysis, elevated liver enzymes and low platelets syndrome; pulmonary oedema; placental abruption; and systemic small vessel spasm. Furthermore, PE substantially increases the risk of adverse pregnancy outcomes, such as foetal growth restriction, premature delivery and stillbirth [5]. There are currently no effective treatments for PE, with premature delivery being almost ineluctable in cases of severe PE. Therefore, identifying risk factors that can help prevent the occurrence of PE is important to reduce morbidity and mortality. The current known risk factors for PE include genetic susceptibility, family history, number of pregnancies, maternal age, maternal smoking, pre-pregnancy body mass index (BMI), the use of assisted reproductive technology and maternal comorbidity condition, such as diabetes, chronic kidney disease and systemic lupus erythematosus [6, 7]. Considering dietary intake can be easily controlled and given, studies on the relationship between dietary factors (energy, nutrients, foods or overall dietary patterns) and PE have attracted more and more attention [8].

The pathophysiology of PE is not clear, but an imbalance between antioxidants and pro-oxidants is known to contribute to the development of the condition [2]. Carotenoids are yellow, orange and red fat-soluble pigments that are widely found in microorganisms, plants, animals and the human body [9]. Carotenoids serve as one of main antioxidant defence systems in the human body [10] and thus play an important role in protection from oxidative stress [11–13]. A recent review noted that the association between carotenoid subclasses and the risk of developing PE has been inconclusive [14]. A meta-analysis of 58 studies, which comprised cohort studies, case–control studies, cross-sectional studies and randomised controlled trials (RCTs), found there was limited and non-definitive evidence that women with PE had low concentrations of carotenoids [15]. Another meta-analysis of RCTs found that oral antioxidant supplements, including lycopene, could not effectively prevent the occurrence of PE [16]. The differences between these meta-analyses’ findings may be partly attributable to differences between the dosages, intervention start times, sample sizes and maternal health status in studies, which demonstrates that more research is required in this field.

Carotenoids can also affect the growth and health of infants, especially their visual and cognitive development [17]. In addition, a recent review recommended that women should consume a diet rich in carotenoids during pregnancy, although there is no current recommendation on carotenoid dietary intake [14]. The few studies that have measured dietary carotenoid intake during pregnancy show that intake varies between study populations [18–20]. There are scarce data from observational studies showing the association between the risk of developing PE and the intake of dietary carotenoids and related compounds, we assessed this relationship in pregnant Chinese women via a hospital-based case–control study.

## Materials and methods

### Study design and participants

This hospital-based case–control study was conducted in the First Affiliated Hospital of Zhengzhou University, Zhengzhou, Henan, China, from March 2016 to June 2019. The inclusion criteria were singleton pregnant woman aged ≥ 18 years and of gestational age ≥  28 weeks. The case group met the diagnostic criteria of China’s diagnosis and treatment guidelines for hypertensive disorders in pregnancy (2015), which have been detailed in our previous studies [21, 22]. The control group was pregnant women from the same hospital with normal blood pressure and no proteinuria. They were matched with case group based on age (± 3 years), gestational age (± 1 weeks) and gestational diabetes mellitus (GDM) status (yes/no). Women with heart disease, malignant tumours, hyperthyroidism, immune system diseases, chronic renal insufficiency, endocrine diseases, mental disorders or those unable to complete the interviews were excluded.

### Assessment of dietary carotenoid intake

A validated semi-quantitative food frequency questionnaire (FFQ) [23] was used to investigate the dietary intake of participants in the 3 months preceding delivery. The FFQ had question in seven categories and regarding a total of 79 food items relevant to Chinese eating habits, including grains, beans and their products, vegetables, fruits, animal foods (meat and poultry, fish and seafood, eggs and dairy), seeds and nuts, and drinks and soups. Participants were asked by trained investigators to report their frequency of consumption of each food item on a daily, weekly or monthly basis during the 3 months preceding delivery. Pre-prepared food pictures were then used to help them estimate the amounts they had consumed. The participants spent 30–40 min recalling their dietary intake with the help of the investigators and these pictures. Their daily intake of energy (kcal/d) and carotenoids (μg/d) was calculated using Chinese Food Composition Tables (2004) [24].

### Data collection

Socio-demographic and lifestyle characteristics were recorded using a structured questionnaire. The daily metabolic equivalent (metabolic equivalents task [MET]) was calculated based on the intensity of average daily physical activity. The height, weight and blood pressure of the participants were measured by trained investigators. Height and weight were accurate to 0.1 cm and 0.1 kg, respectively. The left-arm blood pressure of the participants was measured at rest at least twice (with an interval of 10 min). If the difference between the first and second systolic blood pressure (SBP) measurement or diastolic blood pressure (DBP) measurement was more than 3 mmHg, a third measurement was performed. The BMI was calculated as weight (kg)/[height (m)]^2^.

### Statistical analysis

Based on previous survey results, we assumed that 25% of the control population consumed more carotenoids and estimated the odds ratio (OR) between higher carotenoid intake and PE to be 0.50 [25]. The minimum sample size of each group was calculated to be 281, based on a statistical power of 90% (β = 0.1) and a two-sided significance level of 5% (α = 0.05). All of the analyses were conducted using SPSS software (v25.0, IBM Corporation) and SAS software (v9.1, SAS Institute Inc.). Two-sided tests were used and *P* <  0.05 was defined as being statistically significant.

To compare the general characteristics and dietary carotenoid intakes of the case and control groups, a paired sample *t*-test or Wilcoxon signed rank-sum test was to analyse quantitative variables and McNemar’s test was used to analyse qualitative variables. To compare the distribution of demographic factors and dietary intake of food groups rich in carotenoids and nutrients according to quartiles of the total carotenoids, ANOVA (*P*_homogeneity of variance test_ > 0.05) and Kruskal-Wallis H test (*P*_homogeneity of variance test_ <  0.05) were used to analyse quantitative variables and Chi square test was used to analyse qualitative variables. Data were described as means and standard deviations (SDs), medians and interquartile range (IQRs), numbers and percentages (%) or mean rank.

Based on the intake data of the control group, the quartile method was used to convert the energy-adjusted dietary carotenoid intake (residual method) [26] of the case group into a categorical variable. The energy-adjusted dietary intake was the residual of the regression of the dietary component’s intake on energy intake. A conditional logistic-regression model was used to examine the association between energy-adjusted dietary carotenoid intake and the risk of developing PE. The potential confounding variables were adjusted in the multivariate model, including age (years), gestational age (weeks), pre-pregnancy BMI (kg/m^2^), gravidity, parity, GDM status (yes/no), family hypertension history (yes/no), physical activity (MET [h/d]), education and income levels and total energy intake (kcal/d). The linear trend was tested by entering the median intake for each quartile of carotenoid intake as a continuous variable into univariate and multivariate logistic regression models. After standardising the energy-adjusted data and entering it into the model, the risk per one SD increase was calculated. A restricted cubic spline (RCS) was used to examine the potentially non-linear relationship between energy-adjusted dietary carotenoid intake and the risk of developing PE (with knots placed on the 20th, 50th and 80th percentiles).

To test the stability of the relationship between the risk of developing PE and the intake of dietary carotenoids and related compounds, a sensitivity analysis was carried out by excluding the GDM participants. Another sensitivity analysis was performed by further adjusted for dietary factors in the logical model on the basis of general confounding factors, including energy-adjusted dietary vegetables, fruits, fat, fibre, vitamin D, vitamin C, vitamin E, and flavonoids intake. In addition, the correlation coefficients between carotenoids and energy intakes were calculated, and the conditional logistic regression using the original data was also performed.

## Results

### Basic characteristics and dietary intakes of total carotenoids and related compounds of case and control group

Four hundred and forty pairs of pregnant women were matched and assessed to explore the relationship between dietary carotenoid intake and the risk of developing PE. The case and control groups’ basic demographic information and data on their intake of total carotenoids and related compounds are listed in Table 1. No significant differences were observed between those the groups in terms of age, gestational age, district, income level and passive smoking status. Compared with the control group, the SBP, DBP, pre-pregnancy BMI and physical activity level of the case group were higher, whereas its total energy intake was lower. Pregnant women with PE were more likely to have a low educational level and family history of hypertension, to be experiencing their first pregnancy and to gain more weight during pregnancy. Those in the case group consumed fewer total carotenoids, α-carotene, β-carotene, β-cryptoxanthin, lycopene and lutein and zeaxanthin (lut-zea) than those in the control group.

**Table 1:** Distribution of demographic factors and energy-adjusted dietary carotenoids compounds intake among cases and controls

	Case Group			Control Group			P*
	n	Mean/Median	SD/IQR	n	Mean/Median	SD/IQR	
Age (years) ^a^	440	30.88	5.03	440	31.03	4.85	0.11
Pregnant age (weeks) ^a^	440	34.17	2.90	440	34.24	2.67	0.07
SBP (mmHg) ^a^	440	153.78	16.71	440	113.17	10.42	< 0.001
DBP (mmHg) ^a^	440	100.21	12.33	440	72.84	8.98	< 0.001
Pre-pregnancy BMI (kg/m^2^) ^a^	440	23.67	3.89	440	22.35	3.35	< 0.001
Physical activity (MET- h/d) ^b^	440	26.17	4.47	440	25.35	5.15	0.03
Total energy intake (kcal/d) ^b^	440	1747.21	622.83	440	1866.97	595.38	0.006
District ^c^							0.864
Zhengzhou	85	19.3		88	20.0		
Others	355	80.7		352	80.0		
Educational level (%) ^c^							0.01
Junior high school and below	207	47.0		165	37.5		
Senior high school	75	17.0		83	18.9		
Undergraduate and above	158	36.0		192	43.6		
Income level (yuan/month) (%) ^c^							0.15
< 3000	185	42.0		166	37.7		
3000 – 6000	194	44.1		193	43.9		
> 6000	61	13.9		81	18.4		
Family hypertension history (%) ^c^							< 0.001
Yes	159	36.1		82	18.6		
No	281	63.9		358	81.4		
Weight-gain during pregnancy (kg) (%) ^c^							< 0.001
< 15	179	40.7		292	66.4		
15–20	142	32.3		122	27.7		
> 20	119	27.0		26	5.9		
Passive smoking (%) ^c^							0.49
Yes	67	15.2		59	13.4		
No	373	84.8		381	86.6		
Gravidity (%) ^c^							0.005
1	116	26.4		79	18.0		
2	112	25.5		105	23.9		
≥ 3	212	48.1		256	58.1		
Parity (%) ^c^							0.001
0	185	42.0		136	30.9		
1–2	249	56.6		286	65.0		
≥ 3	6	1.4		18	4.1		
Total carotenoids intake (μg/d) ^b^	440	14,532.38	8108.91	440	17,088.73	9849.94	< 0.001
α-carotene intake (μg/d) ^b^	440	815.59	750.47	440	920.02	908.30	0.006
β-carotene intake (μg/d) ^b^	440	6138.40	3474.44	440	7188.93	4422.15	< 0.001
β-cryptoxanthin intake (μg/d) ^b^	440	86.75	71.94	440	101.07	102.00	< 0.001
Lycopene intake (μg/d) ^b^	440	2178.33	2377.52	440	2931.10	3566.79	< 0.001
Lut-zea intake (μg/d) ^b^	440	4418.76	3372.54	440	5069.52	3386.23	< 0.001

*SD* Standard deviation, *IQR* Interquartile range, *SBP* Systolic blood pressure, *DBP* Diastolic blood pressure, *BMI* Body mass index, *MET* Metabolic equivalent task, *Lut-zea* Lutein and zeaxanthin

^a^ Described as means and SDs. ^b^ Described as medians and IQRs. ^c^ Described as numbers and percentages

*Paired sample *t*-test or Wilcoxon signed rank-sum test was used to analyse quantitative variables and McNemar’s test was used to analyse qualitative variables

### General characteristics and dietary intakes of the participants by quartiles of total carotenoids

The distribution of demographic factors and dietary intake of food groups rich in carotenoids and nutrients according to quartiles of the total carotenoids are listed in Table 2. The results showed that dietary carotenoids intake was related to age, energy-adjusted dietary vegetables, fruits, fat, protein, fibre, vitamin D, vitamin C and flavonoids intake (*P* <  0.05).

**Table 2:** Distribution of demographic factors and dietary intake of food groups rich in carotenoids and nutrients according to quartiles of the energy-adjusted consumption of total carotenoids

	Total carotenoids (mg/d)												P^*^
	Q_1_ (< 11.88 mg/d)			Q_2_ (11.88–15.98 mg/d)			Q_3_ (15.99–20.78 mg/d)			Q_4_ (> 20.78 mg/d)			
	n	Mean/Median	SD/IQR	n	Mean/Median	SD/IQR	n	Mean/Median	SD/IQR	n	Mean/Median	SD/IQR	
Age (years) ^a^	220	30.2	5.01	220	30.6	4.93	220	30.99	4.95	220	32.04	4.69	0.001
Pregnant age (weeks) ^a^	220	34.20	2.84	220	34.33	2.77	220	33.96	2.74	220	34.33	2.80	0.47
SBP (mmHg) ^a^	220	136.47	24.55	220	135.09	24.43	220	133.18	25.44	220	129.26	23.88	0.02
DBP (mmHg) ^a^	220	87.76	16.69	220	87.45	17.32	220	87.15	18.32	220	83.73	17.29	0.06
Pre-pregnancy BMI (kg/m^2^)^a^	220	23.27	3.72	220	22.47	3.53	220	23.11	3.84	220	23.18	3.63	0.09
Physical activity (MET- h/d)^a^	220	26.49	4.21	220	26.95	4.30	220	27.00	4.25	220	26.65	4.14	0.52
District (%)^b^													0.366
Zhengzhou	50	18.9		43	18.8		40	19.0		40	22.9		
Others	215	81.1		186	81.2		171	81.0		135	77.1		
Educational level (%)^b^		0.15											0.15
Junior high school and below	106	48.2		92	41.8		86	39.1		87	39.5		
Senior high school	45	20.5		38	17.3		36	16.4		40	18.2		
Undergraduate and above	69	31.3		90	40.9		98	44.5		93	42.3		
Income level (yuan/month) (%) ^b^													0.34
< 3000	96	43.6		83	37.7		76	34.5		81	36.8		
3000 – 6000	90	40.9		90	40.9		92	41.8		90	40.9		
> 6000	34	15.5		47	21.4		52	23.6		49	22.3		
Family hypertension history (%) ^b^													0.57
Yes	60	27.3		61	27.7		69	31.4		56	25.5		
No	160	72.7		159	72.3		151	68.6		164	74.5		
Weight-gain during pregnancy (kg) (%) ^b^													0.09
< 15	112	50.9		109	49.5		120	54.5		130	59.1		
15–20	62	28.2		75	34.1		61	27.7		66	30.0		
> 20	46	20.9		36	16.4		39	17.7		24	10.9		
Passive smoking (%) ^b^													0.43
Yes	126	57.3		117	53.2		110	50.0		112	50.9		
No	94	42.7		103	46.8		110	50		108	49.1		
Gravidity (%) ^b^													0.09
1	57	25.9		56	25.5		48	21.8		34	15.5		
2	50	22.7		48	21.8		62	28.2		57	25.9		
≥ 3	113	51.4		116	52.7		110	50.0		129	58.6		
Parity (%) ^b^													0.32
0	88	40.0		85	38.6		83	37.7		67	30.5		
1–2	126	57.3		128	58.2		134	60.9		146	66.4		
≥ 3	6	2.7		7	3.2		3	1.4		7	3.2		
Total energy intake (kcal/d) ^c^	220	464.37		220	430.56		220	408.45		220	458.62		0.08
Energy-adjusted dietary vegetables intake (g/d) ^c^	220	201.04		220	394.41		220	503.60		220	662.95		< 0.001
Energy-adjusted dietary fruits intake (g/d) ^c^	220	390.10		220	392.92		220	439.73		220	539.25		< 0.001
Energy-adjusted dietary carbohydrates intake (g/d) ^c^	220	439.67		220	427.81		220	432.39		220	462.14		0.50
Energy-adjusted dietary fat intake (g/d) ^c^	220	458.84		220	464.85		220	443.82		220	394.49		0.02
Energy-adjusted dietary protein intake (g/d) ^c^	220	360.03		220	402.76		220	457.04		220	542.17		< 0.001
Energy-adjusted dietary fiber intake (g/d) ^c^	220	277.45		220	365.78		220	458.93		220	659.84		< 0.001
Energy-adjusted dietary vitamin D intake (μg/d) ^c^	220	395.29		220	410.73		220	465.66		220	490.32		< 0.001
Energy-adjusted dietary vitamin C intake (mg/d) ^c^	220	245.01		220	360.40		220	484.50		220	672.09		< 0.001
Energy-adjusted dietary vitamin E intake (mg/d) ^a^	220	30.16	9.58	220	31.79	10.07	220	32.07	9.05	220	31.05	8.44	0.14
Energy-adjusted dietary flavonoids intake (mg/d) ^c^	220	392.40		220	417.63		220	445.64		220	506.33		< 0.001

*SD* Standard deviation, *IQR* Interquartile range, *SBP* systolic blood pressure, *DBP* Diastolic blood pressure, *BMI* Body mass index, *MET* Metabolic equivalent task, *Lut-zea* Lutein and zeaxanthin

^a^ Described as means and SDs. ^b^ Described as numbers and percentages. ^c^ Described as mean rank

*ANOVA (*P*_homogeneity of variance test_ > 0.05) and Kruskal-Wallis H test (*P*_homogeneity of variance test_ < 0.05) were used to analyse quantitative variables and Chi square test was used to analyse qualitative variables

### Association between intake of dietary carotenoids and their subclasses and the risk of PE

The results of conditional logistic regression are shown in Table 3. A comparison of the highest and lowest intake quartiles shows that the consumption of total carotenoids, α-carotene, β-carotene, β-cryptoxanthin, lycopene and lut-zea were negatively associated with the risk of developing PE. After adjusting for all of the potential confounders, we found that high intakes of total carotenoids, β-carotene, β-cryptoxanthin, lycopene and lut-zea were associated with a lower risk of developing PE. Compared with the lowest quartile intake, the multivariate-adjusted OR (95% confidence interval [CI]) of the highest quartile intake was 0.29 (0.16–0.54, *P*_trend_ <  0.001) for total carotenoids, 0.31(0.16–0.58, *P*_trend_ <  0.001) for β-carotene, 0.50 (0.27–0.90, *P*_trend_ = 0.007) for β-cryptoxanthin, 0.55 (0.30–0.99, *P*_trend_ = 0.04) for lycopene and 0.32 (0.17–0.61, *P*_trend_ = 0.001) for lut-zea. However, there were no associations observed between PE and α-carotene intake (OR = 0.75, 95% CI: 0.41–1.36, *P*_trend_ = 0.28). Moreover, the odds of developing PE decreased by 38% (OR = 0.62, 95% CI: 0.48–0.79), 28% (OR = 0.72, 95% CI: 0.58–0.89), 33% (OR = 0.67, 95% CI: 0.47–0.95), 31% (OR = 0.69, 95% CI: 0.53–0.90) and 33% (OR = 0.67, 95% CI: 0.53–0.85) for every one SD increase in consumption of total carotenoids, β-carotene, β-cryptoxanthin, lycopene and lut-zea, respectively.

**Table 3:** Association between energy-adjusted intake of dietary carotenoids and their subclasses and the risk of developing PE

	Quartiles of carotenoid intakes (*OR*, 95% *CI*)				*P*_trend_^a^	Per one-SD increase ^b^
	Q_1_	Q_2_	Q_3_	Q_4_		
Total carotenoids						
Crude	1.0	0.76 (0.54, 1.08)	0.65 (0.45, 0.94)*	0.41 (0.27, 0.61)**	< 0.001	0.67 (0.57, 0.78)**
Adjusted model	1.0	0.55 (0.31, 0.96)*	0.52 (0.28, 0.97)*	0.29 (0.16, 0.54)**	< 0.001	0.62 (0.48, 0.79)**
α-carotene						
Crude	1.0	1.04 (0.73, 1.48)	0.96 (0.67, 1.38)	0.69 (0.45, 0.99)*	0.03	0.88 (0.77, 1.01)
Adjusted model	1.0	0.88 (0.51, 1.52)	0.93 (0.52, 1.68)	0.75 (0.41, 1.36)	0.28	0.95 (0.79, 1.15)
β-carotene						
Crude	1.0	0.75 (0.52, 1.08)	0.68 (0.47, 0.99)*	0.47 (0.32, 0.70)**	< 0.001	0.74 (0.63, 0.85)**
Adjusted model	1.0	0.58 (0.33, 1.03)	0.38 (0.21, 0.71)*	0.31 (0.16, 0.58)**	< 0.001	0.72 (0.58, 0.89)*
β-cryptoxanthin						
Crude	1.0	0.97 (0.68, 1.39)	0.87 (0.59, 1.29)	0.70 (0.47, 1.03)	0.04	0.72 (0.58, 0.88)*
Adjusted model	1.0	1.19 (0.68, 2.09)	0.94 (0.51, 1.75)	0.50 (0.27, 0.90)*	0.007	0.67 (0.47, 0.95)*
Lycopene						
Crude	1.0	0.99 (0.68, 1.44)	0.74 (0.50, 1.08)	0.52 (0.35, 0.77)*	< 0.001	0.64 (0.53, 0.77)**
Adjusted model	1.0	0.69 (0.39, 1.21)	0.61 (0.33, 1.12)	0.55 (0.30, 0.99)*	0.04	0.69 (0.53, 0.90)*
Lut-zea						
Crude	1.0	0.86 (0.61, 1.23)	0.65 (0.43, 0.96)*	0.59 (0.39, 0.87)*	0.004	0.80 (0.69, 0.93)*
Adjusted model	1.0	0.58 (0.32, 1.06)	0.48 (0.26, 0.90)*	0.32 (0.17, 0.61)**	0.001	0.67 (0.53, 0.85)*

*PE* Preeclampsia, *OR* odds ratio, *CI* confidence interval, *Q* Quartile, *SD* Standard deviation, *Lut-zea* Lutein and zeaxanthin

^a^ Tested by entering the median intake of each quartile of carotenoids as a continuous variable into univariate and multivariate logistic regression models. ^b^ Performed by standardising the energy-adjusted carotenoid intakes data and entering it into the model.

Adjusted model was adjusted for age (years), gestational age (weeks), pre-pregnancy BMI (kg/m^2^), gravidity, parity, gestational diabetes mellitus (GDM) status (yes/no), family hypertension history (yes/no), physical activity (MET [h/d]), education and income levels and total energy intake (kcal/d)

*P < 0.05; **P < 0.001.

### Multivariable-adjusted RCS analyses

**Fig. 1:**
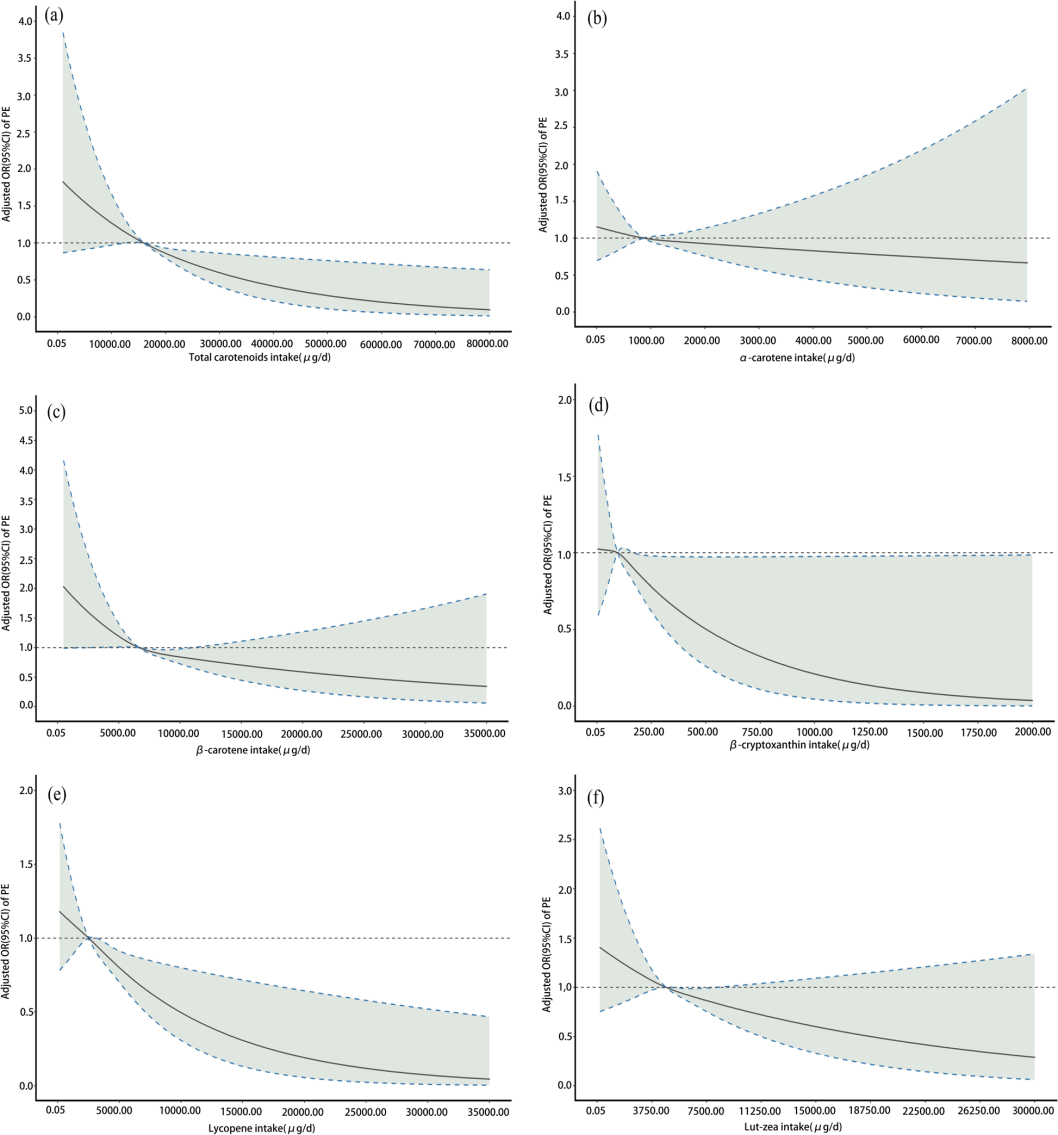
Multivariable-adjusted spline curve of association between energy-adjusted dietary carotenoid intake and the risk of developing PE. Multivariable-adjusted odds ratios (ORs) (solid lines) and 95% confidence intervals (CIs) (dashed lines) for risk of developing PE based on the dietary intake of total carotenoids (**a**), α-carotene (**b**), β-carotene (**c**), β-cryptoxanthin (**d**), lycopene (**e**) and lutein-zeaxanthin (lut-zea) (**f**). The horizontal dashed line represents an OR of 1. The ORs were adjusted for age (years), gestational age (weeks), pre-pregnancy BMI (kg/m^2^), total energy intake (kcal/d), gravidity, parity, gestational diabetes mellitus (GDM) status (yes/no), family hypertension history (yes/no), physical activity (MET [h/d]) and education and income level

The results of the multivariable-adjusted RCS analysis are shown in Fig. 1. Broadly negative associations were observed between the consumption of total carotenoids (*P*-overall association < 0.001, *P*-nonlinearity = 0.91), β-carotene (*P*-overall association = 0.01, *P*-nonlinearity = 0.32) and lycopene (*P*-overall association = 0.004, *P*-nonlinearity = 0.82) and the risk of developing PE.

### Sensitivity analysis

(1) Data on the pregnant women with GDM were removed, and a sensitivity analysis was performed on the remaining data of 382 pairs of women (Table 4). Overall, similar results were found after adjusting for the confounders.

**Table 4:** Sensitivity analysis after excluding GDM patients

	Quartiles of energy-adjusted carotenoid intakes (*OR*, 95% *CI*)				*P*_trend_^a^	Per one-SD increase ^b^
	Q_1_	Q_2_	Q_3_	Q_4_		
Total carotenoids						
Crude	1.0	0.75 (0.51, 1.09)	0.61 (0.41, 0.90)*	0.49 (0.32, 0.75)**	0.001	0.71 (0.60, 0.84)**
Adjusted model	1.0	0.57 (0.31, 1.03)	0.47 (0.24, 0.90)*	0.36 (0.18, 0.69)**	0.002	0.65 (0.51, 0.84)**
α-carotene						
Crude	1.0	1.09 (0.74, 1.60)	0.93 (0.63, 1.37)	0.71 (0.46, 1.09)	0.06	0.91 (0.79, 1.05)
Adjusted model	1.0	0.89 (0.49, 1.59)	0.87 (0.47, 1.62)	0.67 (0.35, 1.30)	0.24	0.94 (0.77, 1.15)
β-carotene						
Crude	1.0	0.72 (0.49, 1.06)	0.63 (0.42, 0.94)*	0.55 (0.36, 0.83)*	0.004	0.78 (0.67, 0.91)*
Adjusted model	1.0	0.61 (0.33, 1.11)	0.37 (0.19, 0.71)*	0.36 (0.18, 0.70)*	0.001	0.76 (0.61, 0.95)*
β-cryptoxanthin						
Crude	1.0	0.92 (0.63, 1.34)	0.93 (0.61, 1.41)	0.75 (0.49, 1.14)	0.18	0.73 (0.59, 0.91)*
Adjusted model	1.0	0.97 (0.54,1.74)	0.79 (0.41, 1.54)	0.49 (0.26, 0.94)*	0.02	0.68 (0.48, 0.98)*
Lycopene						
Crude	1.0	1.01 (0.68, 1.51)	0.81 (0.54, 1.21)	0.59 (0.39, 0.90)*	0.006	0.69 (0.57, 0.84)**
Adjusted model	1.0	0.69 (0.38, 1.25)	0.58 (0.30, 1.10)	0.61 (0.32, 1.14)	0.14	0.70 (0.53, 0.93)*
Lut-zea						
Crude	1.0	0.81 (0.56, 1.18)	0.62 (0.40, 0.94)*	0.59 (0.39, 0.91)*	0.01	0.82 (0.69, 0.96)*
Adjusted model	1.0	0.59 (0.31, 1.10)	0.49 (0.25, 0.95)*	0.35 (0.18, 0.69)*	0.003	0.67 (0.51, 0.87)*

*GDM* Gestational diabetes mellitus, *OR* Odds ratio, *CI* Confidence interval, *Q* Quartile, *SD* Standard deviation, *Lut-zea* Lutein and zeaxanthin

^a^ Tested by entering the median intake of each quartile of carotenoids as a continuous variable into univariate and multivariate logistic regression models. ^b^ Performed by standardising the energy-adjusted carotenoid intakes data and entering it into the model.

Adjusted model was adjusted for age (years), gestational age (weeks), pre-pregnancy BMI (kg/m^2^), gravidity, parity, gestational diabetes mellitus (GDM) status (yes/no), family hypertension history (yes/no), physical activity (MET [h/d]), education and income levels and total energy intake (kcal/d)

*P < 0.05; **P < 0.001.

(2) The results of further adjusted for the dietary factors also showed consistency with our earlier findings (Table 5).

**Table 5:** Sensitivity analysis of further adjusting dietary confounding factors on the basis of the general confounders

	Quartiles of energy-adjusted carotenoid intakes (*OR*, 95% *CI*)				*P*_trend_^a^	Per one-SD increase ^b^
	Q_1_	Q_2_	Q_3_	Q_4_		
Total carotenoids	1.0	0.47 (0.25, 0.87)*	0.44 (0.22, 0.87)*	0.23 (0.12, 0.46)**	< 0.001	0.60 (0.47, 0.77)**
α-carotene	1.0	0.76 (0.42, 1.38)	0.75 (0.40, 1.40)	0.66 (0.35, 1.26)	0.24	0.94 (0.78, 1.14)
β-carotene	1.0	0.59 (0.32, 1.08)	0.36 (0.19, 0.68)*	0.24 (0.12, 0.49)**	< 0.001	0.68 (0.55, 0.86)*
β-cryptoxanthin	1.0	1.11 (0.62, 2.01)	0.82 (0.42, 1.58)	0.54 (0.29, 0.99)*	0.02	0.77 (0.60, 0.99)*
Lycopene	1.0	0.73 (0.40, 1.33)	0.68 (0.36, 1.29)	0.55 (0.29, 1.04)	0.09	0.74 (0.58, 0.93)*
Lut-zea	1.0	0.51 (0.26, 0.97)*	0.40 (0.20, 0.80)*	0.25 (0.12, 0.51)**	< 0.001	0.64 (0.50, 0.83)*

^a^ Tested by entering the median intake of each quartile of carotenoids as a continuous variable into univariate and multivariate logistic regression models. ^b^ Performed by standardising the energy-adjusted carotenoid intakes data and entering it into the model.

Adjustment for age (years), gestational age (weeks), pre-pregnancy BMI (kg/m^2^), gravidity, parity, gestational diabetes mellitus (GDM) status (yes/no), family hypertension history (yes/no), physical activity (MET [h/d]), education and income levels, total energy intake (kcal/d) and dietary factors (energy-adjusted dietary vegetables/ fruits/ fat/ fiber/ vitamin D/ vitamin C/ vitamin E/ flavonoids intake)

*P < 0.05; **P < 0.001.

(3) The correlation coefficients between carotenoids and energy intakes were 0.196 for α-carotene, 0.313 for β-carotene, 0.379 for β-cryptoxanthin, 0.255 for lycopene, 0.231 for lut-zea and 0.337 for total carotenoids. The conditional logistic regression using the original data was performed, yet similar results were found (Additional file 1).

## Discussion

To the best of our knowledge, this was the first study to assess the relationship between the risk of developing PE and the intake of dietary carotenoids and related compounds in China. After adjusting for potential confounding factors, we found that a high intake of total carotenoids, β-carotene, β-cryptoxanthin, lycopene and lut-zea was associated with a reduced risk of developing PE. The highest quartile intakes of dietary carotenoids, except that of α-carotene, were associated with a 45% or greater reduction in the risk of developing PE than the other quartiles, which indicated a significant dose-response trend. The carotenoid profiles were considered to be relatively complete in this study, and represented approximately 95% of the carotenoids in the blood [27]. We included six of the most studied categories: α-carotene, β-carotene, β-cryptoxanthin, lycopene, and lut-zea.

Our results for total carotenoids, α-carotene, β-carotene and lutein were consistent with those of several previous studies that have reported that the total carotenoid concentrations in the sera of patients with mild and severe PE were significantly lower than those in the sera of healthy pregnant women [28, 29]. Our results for β-carotene were similar to those of a case–control study conducted by Yusuf, et al. [30] in Jordan, which used three 24-h dietary records to compare dietary carotenoid intake of a PE and a control groups. It should be noted that only correlation analysis (Chi-square test) was performed in their study, but further correlation strength (OR) analysis was not. In addition, dietary recording may lead to changes in eating behavior in the process of recording food intake, suggesting that bias may exist. In contrast, FFQ covers a longer time span, so it may better reflect habitual intake than dietary record, and may be more suitable for epidemiological studies on the relationship between dietary exposure and chronic diseases. A case–control study conducted in Zimbabwe (*n* = 173/186) showed that higher concentrations of β-carotene in the blood were inversely associated with the risk of developing PE [25]. Cohen et al. [31] found an inverse association between lycopene, lutein and total carotenoid concentrations and the risk of developing PE, but lutein was the only carotenoid that was significantly associated after adjusting for confounders. Zhang et al. [32] did not find any relationships between plasma α-carotene concentrations and the risk of developing PE.

The current study has some inconsistencies with previous studies on β-cryptoxanthin and lycopene. For example, Zhang et al. [32] did not find any associations between the risk of developing PE and the intake of β-cryptoxanthin and lycopene (*n* = 125/179). Notably, the blood samples in that study were collected only when the clinical manifestations of PE became obvious. In addition, the gestational age at the time of blood collection from the case and control groups in their study was different (36.0 ± 0.3 weeks vs. 37.3 ± 0.3 weeks, *P* <  0.05). Similarly, two RCTs [33, 34] that have been conducted in India did not find that lycopene intake reduced the incidence rate of PE (*n* = 44/54 and 159/159, respectively). However, in the current study, we found that a high lycopene intake had an unambiguously protective effect against the development of PE (lycopene intakes in the case and control groups were 2.18 mg and 2.93 mg, respectively). Our results are similar to those of another RCT (*n* = 116/135) conducted in India [35], which found that fewer pregnant women developed PE in the intervention group supplemented with 4 mg lycopene per day from 16 to 20 weeks compared to the placebo group (intervention vs. placebo: 8.6% vs. 17.7%, *P* = 0.04). Subsequently, blood samples from 50 PE pregnant women and 50 healthy pregnant women in this Indian study revealed that those with PE had significantly lower blood concentrations of lycopene than those without PE [36]. Finally, a meta-analysis conducted in 2018 of RCTs of oral antioxidant therapy for the prevention and treatment of PE found that oral lycopene supplementation of 2 mg/d or 4 mg/d from the second trimester to delivery did not prevent the occurrence of PE (relative risk [RR] = 0.67, 95% CI: 0.44–1.04, *P* = 0.08) [16]. Many factors could be responsible for these differences between study results, such as the study design, the treatment dose, the season, and pregnant women’s ethnicity and health status (high or low PE risk). Previous studies have found that pregnant women who are younger [12], smoking [19], and getting pregnant in the spring and summer [20] consume fewer carotenoids. In view of the results of this current study, future large-scale studies are required on the dietary carotenoid intake of pregnant women with PE.

Although most of the studies only measured blood concentrations and did not collect dietary data, they indirectly confirm our results, as the human body cannot synthesise carotenoids and thus the carotenoids in blood are derived from the diet. The Norwegian Mother and Child Cohort Study (MoBa) found that the plasma carotenoid concentrations of pregnant women were significantly correlated with food consumption, especially of fruits (r = 0.24; *p* <  0.01) and vegetables (r = 0.32; p <  0.01), which lends validity to our method [37]. In their study, strong correlations between carotenoids and food intake were found between a-carotene and carrots (r = 0.50; *p* < 0.01) and cooked vegetables (r = 0.39; p < 0.01). A meta-analysis [38] of RCTs on the effect of changes in fruit and vegetable intake on carotenoid blood concentration showed that four common carotenoids (α-carotene, β-carotene, β-cryptoxanthin and lutein) may be used as biomarkers to objectively measure general fruit and vegetable intake.

Currently, the pathogenesis of PE is thought to involve two stages: reduced placental perfusion and secondary maternal multisystem impairment [2]. Placental ischaemia and reperfusion injury caused by insufficient remodelling of the uterine spiral artery is considered to be a cause of oxidative stress [39–41], which has also been confirmed by animal [42] and in vitro experiments [43]. The injury of vascular endothelial function caused by oxidative stress and inflammatory media leads to the decrease of vasodilators and the increase of vasoconstrictors, thus promoting vasospasm [44, 45]. It is clear that oxidative stress, a basic pathophysiological mediator of PE, plays an important role in the occurrence of PE. In truth, oxidative stress plays a key role in the development of both normal and defective placentas [2, 46]. However, there appears to be an imbalance between antioxidant and pro-oxidant mechanisms in PE. Some studies have confirmed that pregnant women with PE have higher concentrations of markers of oxidative damage and lower concentrations of antioxidants and lower total antioxidant capacity than healthy pregnant women [36, 47, 48]. The human body has various antioxidants, both enzymatic and non-enzymatic, to counter the effects of oxidants. Among non-enzymatic antioxidants, nutrients such as vitamin C, vitamin E and carotenoids play a crucial role in the human antioxidant system [10]. Previous studies have found that the intake of antioxidant nutrients affects oxidative stress [49, 50], with an insufficient intake increasing the risk of higher levels of oxidative stress and leading to poor obstetric outcomes [51, 52]. Two Cochrane reviews evaluated the effectiveness of vitamin C and E alone or in combination in the prevention of PE, but no benefit was found for routine supplementation of vitamin C and/or E [53, 54]. Carotenoids contain many double bonds that can quench singlet oxygen and scavenge free radicals and oxides, which gives them significant antioxidant activity [27]. The demand for micronutrients becomes more sensitive during pregnancy and inadequate intake may affect the mother and foetus [55]. Therefore, it is important to fully understand the role of dietary carotenoids in the occurrence of PE.

In addition to antioxidant nutrients, vitamin D supplementation has been proposed for the prevention of PE, although evidence has been inconsistent [56]. Our previous research observed a negative relationship between dietary VD intake and PE risk [57]. Vitamin D is a regulator of inflammation and may affect the occurrence of PE by regulating maternal immune response and reducing the concentrations of proinflammatory cytokines. Furthermore, Yusuf, et al. [30] found a strong positive association exists for the intake of fat and negative association for the vegetables and fruits with PE. A systematic literature review and meta-analysis confirmed that high intake of fruits and vegetables had a protective effect on PE [8]. Brantsæter et al. [58] found that “vegetable dietary pattern” characterized by vegetables, plant-based foods and vegetable oils could reduce the risk of PE, whereas a dietary pattern characterized by a high consumption of processed meat, sweet drinks, and salty snacks increases the likelihood of PE. The possible reason is plant foods are rich in micronutrients (phytochemicals, antioxidants, vitamins and minerals) and dietary fibre. Moreover, a review pointed out that high intake of carotenoids may be related to a healthier diet and lifestyle, which may be beneficial in themselves [14]. Considering the role of these dietary related factors, a sensitivity analysis was performed on the basis of general confounding factors. But the results indicated that a high intake of total carotenoids, β-carotene, β-cryptoxanthin and lut-zea was still associated with a reduced risk of developing PE, which may reflect that carotenoids intake can play a role as a predictor in the risk of PE, independent of the effects of other nutritional factors.

Some limitations of the study should be acknowledged. First, the causality of the studied association remained uncertain due to the inherent limitation of the retrospective study design of case–control study. Second, according to the rare disease assumption in the case-control study, OR might not well estimate RR (relative ratio) for the low incidence (but not rare) disease of PE in China (about 3%) [59]. The First Affiliated Hospital of Zhengzhou University is the most authoritative comprehensive hospital in Henan Province and has treated enough PE patients in this area. We recruited almost all incident cases which admitted in the target hospitals on workdays, but some cases admitted on holidays might be missed. For hospital-based case-control studies, it’s really hard to exactly identify the source population of the cases and controls. We compared the distribution of the source of the study population (Zhengzhou and other regions) in the case group and the control group, and found that the proportion of the two groups from Zhengzhou was about 20%, suggesting that the two groups were comparable. Thus, in this case, we believe OR may be an acceptable choice for estimating of the incidence rate ratio. Third, the data on dietary carotenoid intake were based on the recall of the participants, which means that recall bias was unavoidable. To minimise recall bias, we took the following measures: (1) participants’ dietary intakes were assessed in face-to-face interviews with trained investigators; (2) detailed food pictures were used to help the participants recall their eating habits; and (3) we investigated dietary intake in only the 3 months immediately preceding delivery, during which period the diet is relatively stable compared to other periods of pregnancy. This meant that our study may be unable to explain the effect of diet in early pregnancy on the occurrence of PE, but given that it is often less than 3 months from the beginning to delivery of PE patients, we believe that this study reflects the relationship between the risk of developing PE and the intake of dietary carotenoids and related compounds to a certain degree. Finally, instead of detecting serum carotenoids, which can be traumatic, low-cooperative and expensive, we investigated dietary carotenoid intake in a non-invasive and low-cost manner using an FFQ. As mentioned above, considering the consistency between dietary and circulating carotenoid levels and the significance of measuring carotenoid levels from dietary sources, this study may guide pregnant women’s dietary behavior and contribute to the prevention of PE.

## Conclusion

Our results indicate that high intakes of total carotenoids, β-Carotene, β-cryptoxanthin, lycopene and lut-zea may be associated with a reduction in the risk of developing PE. Larger-scale studies are needed to verify this relationship. In the future, we recommend that pregnant women consume carotenoid-rich foods during pregnancy to prevent the occurrence of PE.

## Supplementary Information

**Additional file 1: Table 1** Association between intake of dietary carotenoids and their subclasses and the risk of developing PE.

## Data Availability

The datasets used and/or analysed during the current study are available from the corresponding author on reasonable request.

## Abbreviations

PE: Preeclampsia
ORs: Odds ratios
Cis: Confidence intervals
Lut-zea: Lutein and zeaxanthin
RCTs: Randomised controlled trials
GDM: Gestational diabetes mellitus
FFQ: Food frequency questionnaire
MET: Metabolic equivalents task
SBP: Systolic blood pressure
DBP: Diastolic blood pressure
BMI: Body mass index
SDs: Standard deviations
IQR: Medians and interquartile range
RCS: Restricted cubic spline
