# Supplementary material for: Dietary carotenoid intake and risk of developing preeclampsia: a hospital-based case–control study
Source: BMC Pregnancy Childbirth. 2022 May 21;22:427. doi: 10.1186/s12884-022-04737-5 (PMC9123797; doi:10.1186/s12884-022-04737-5)
Supplement: Supplementary file 1 — Additional file 1: Table 1 Association between intake of dietary carotenoids and their subclasses and the risk of developing PE. [file 12884_2022_4737_MOESM1_ESM.docx]

**Additional Table 1** Association between intake of dietary carotenoids and their subclasses and the risk of developing PE

|  | Quartiles of carotenoid intakes（*OR*, 95% *CI*） | | | | *P*_trend_ ^a^ | Per one-SD increase ^b^ |  |
| --- | --- | --- | --- | --- | --- | --- | --- |
|  | Q_1_ | Q_2_ | Q_3_ | Q_4_ |  |  |  |
| Total carotenoids |  | | | | | |  |
| Crude | 1 | 0.77  (0.53, 1.11) | 0.49***  (0.33, 0.72) | 0.34***  (0.22, 0.51) | < 0.001 | 0.67***  (0.57, 0.78) | |
| Adjusted model | 1 | 0.62  (0.34, 1.14) | 0.40**  (0.21, 0.80) | 0.24***  (0.13, 0.48) | 0.002 | 0.61***  (0.48, 0.78) | |
| α-carotene |  | | | | | | |
| Crude | 1 | 1.12  (0.79, 1.59) | 0.83  (0.57, 1.21) | 0.68  (0.46, 1.01) | 0.01 | 0.88  (0.77, 1.01) | |
| Adjusted model | 1 | 1.21  (0.69, 2.11) | 1.07  (0.58, 1.99) | 0.81  (0.44, 1.51) | 0.35 | 0.96  (0.79, 1.16) | |
| β-carotene |  | | | | | | |
| Crude | 1 | 0.81  (0.55, 1.18) | 0.48**  (0.33, 0.70) | 0.44**  (0.29, 0.65) | < 0.001 | 0.74***  (0.63, 0.85) | |
| Adjusted model | 1 | 0.69  (0.38, 1.25) | 0.32**  (0.17, 0.61) | 0.29***  (0.15, 0.55) | < 0.001 | 0.73**  (0.58, 0.90) | |
| β-cryptoxanthin |  | | | | | | |
| Crude | 1 | 0.94  (0.66, 1.34) | 0.78  (0.53, 1.13) | 0.53**  (0.35, 0.79) | 0.001 | 0.72**  (0.58, 0.88) | |
| Adjusted model | 1 | 0.98  (0.54, 1.76) | 0.73  (0.40, 1.31) | 0.45*  (0.23, 0.88) | 0.01 | 0.70*  (0.50, 0.99) | |
| Lycopene |  | | | | | | |
| Crude | 1 | 0.87  (0.61, 1.24) | 0.60**  (0.41, 0.88) | 0.45***  (0.30, 0.67) | < 0.001 | 0.64***  (0.53, 0.77) | |
| Adjusted model | 1 | 0.84  (0.47, 1.49) | 0.61  (0.33, 1.13) | 0.51*  (0.27, 0.94) | 0.02 | 0.69**  (0.53, 0.91) | |
| Lut-zea |  | | | | | | |
| Crude | 1 | 0.98  (0.69, 1.39) | 0.57**  (0.38, 0.84) | 0.64*  (0.44, 0.95) | 0.004 | 0.80**  (0.70, 0.93) | |
| Adjusted model | 1 | 1.27  (0.69, 2.34) | 0.52*  (0.27, 0.99) | 0.56  (0.29, 1.09) | 0.006 | 0.65**  (0.51, 0.84) | |

PE, preeclampsia; OR, odds ratio; CI, confidence interval; Q, quartile; SD, standard deviation; Lut-zea, lutein and zeaxanthin.

^a^ Tested by entering the median intake of each quartile of carotenoids as a continuous variable into univariate and multivariate logistic regression models. ^b^ Performed by standardising the original data and entering it into the model.

Adjusted model was adjusted for age (years), gestational age (weeks), pre-pregnancy BMI (kg/m^2^), gravidity, parity, gestational diabetes mellitus (GDM) status (yes/no), family hypertension history (yes/no), physical activity (MET [h/d]), education and income levels and total energy intake (kcal/d).

**P* < 0.05; ***P* < 0.01; ****P* < 0.001.
